# Progress on Multifunction Enzyme-Activated Organic Fluorescent Probes for Bioimaging

**DOI:** 10.3389/fchem.2022.935586

**Published:** 2022-07-13

**Authors:** Jie Lian, Yipeng Wang, Xiaomeng Sun, Quanshi Shi, Fanda Meng

**Affiliations:** ^1^ College of Criminal Investigation, People’s Public Security University of China, Beijing, China; ^2^ School of Clinical and Basic Medical Sciences, Shandong First Medical University & Shandong Academy of Medical Sciences, Jinan, China; ^3^ Department of Clinical Laboratory Medicine, The First Affiliated Hospital of Shandong First Medical University & Shandong Provincial Qianfoshan Hospital, Shandong Medicine and Health Key Laboratory of Laboratory Medicine, Jinan, China; ^4^ Department of Burns and Plastic Surgery, Zaozhuang Hospital of Shandong Healthcare Industry Development Group, Zaozhuang, China

**Keywords:** multifunction, enzyme-activated fluorophore, AIE fluorophore, high selectivity, real-time imaging

## Abstract

Bioimaging techniques are of increasing importance in clinical and related fields, which also have been successfully applied in the *in vivo*/*in vitro* imaging system. Due to the vital factor of enzymes in biological systems, enzyme-activated fluorophores, which could turn “on” the fluorescence signal from an “off” state, offer non-invasive and effective potential for the accurate bioimaging of particular cells, tissues, or bacteria. Comparing with the traditional imaging probes, enzyme-activated organic small fluorophores can visualize living cells within small animals with high sensitivity, high imaging resolution, non-invasiveness, and real-time feedback. In this mini review, well-designed enzyme-activated organic fluorescent probes with multiple functions are exclusively reviewed through the latest development and progress, focusing on probe design strategy, fluorescence property, enzyme activation process, and bioimaging applications. It is worth noting that multi-enzyme-activated strategies, which could avoid the production of “false-positive” signals in complex biological systems, effectively provide high selective and real-time bioimaging, indicating the exciting potential of intraoperative fluorescence imaging and diagnosis tools.

## 1 Introduction

Modern bioimaging techniques are of increasing importance in research and practice spanning basic biochemical studies to clinical applications (Fujibayashi et al., 2002; Miyawaki and Niino, 2015; Salditt et al., 2020), even in forensic practice, where such imaging techniques have outstanding multiple advantages with digital data for multiple reviewers and easily understandable three-dimensional images. Clinic and research bioimaging techniques include X-ray fluoroscopy, X-ray computed tomography (CT), ultrasound imaging, magnetic resonance imaging (MRI), positron emission tomography (PET), single-photon emission computed tomography (SPECT), photoacoustic imaging (PAI), fluorescence imaging (FL), and so on. Among these techniques, fluorescence technology progressed from microscopy, cell, and animal studies, even to clinical practice in several disease areas. Nowadays, fluorescence imaging has been widely used and studied both in cell imaging and *in vivo* imaging system, due to simple operation, high-resolution, non-invasive, and real-time imaging (Frangioni, 2003; Vonesch et al., 2006; Miyawaki and Niino, 2015). Fluorescence imaging could qualitatively and quantitively visualize and characterize biological processes with the wavelength range generally in the visible infrared region, permitting optimal penetration of light through skin and tissue. Previously developed fluorophores that are activated inside the cells have the limitation that, once turned on, they continue to fluoresce wherever they are, making it difficult to track aimed vital tissues. At present, many fluorophores have been commercialized for dyeing the biological functional molecules or various organelles in cells, continuously tracking the selected molecule *in vivo* and recording the physiological process in detail (Hong et al., 2017; Schouw et al., 2021). Due to the combination of targeting ligands and fluorophores, these fluorescence imaging systems also show unique advantages in the accuracy with which specified molecules are tracked. In combination with targeting ligands, disease and vital tissue can be detected with targeted fluorophores, resulting in targeted therapy, image-guided surgery, and personalized medicine. As human vision could not see under the tissue surface and operates on low contrast between sites of disease and the surrounding tissue, intraoperative fluorescence imaging is emerging to improve surgical vision and offers the potential to be integrated as a highly effective real-time imaging and theragnostic tools in the operating room.

Major research efforts have been undertaken to create activated fluorescent probes with high resolution and selectivity. Experimentally, the logical workflow for developing an effective activated fluorescent probe is the structure optimization experiment and test imaging performance in cell culture or in animal models. Compared with traditional probes, organic small fluorophores with high structural flexibility promises higher contrast, sensitivity, and penetration depths. Activatable organic fluorophores are of ongoing research interest as bioimaging contrast agents, for their low background and high specificity to the imaging target as well as quick diffusion and deep penetration into living cells. A typically activated organic fluorophore is a covalently linked conjugate composed of the fluorochromes and activatable targeting ligands, such as antibodies, glycoprotein ligands, aptamers, and short peptide sequences (Urano et al., 2011; Kocaoglu and Carlson, 2016; Kim et al., 2017).

Enzymes, produced by living cells, act as the most powerful catalysts and play an important role in living organisms with catalytic function. The abnormal changes of enzymes are related to a variety of diseases, so enzymes are always used as important biomarkers in the selective detection, early diagnosis, and effective treatment of diseases. With the targeting ligands as enzyme substrates, enzyme-activated fluorophore, the smart fluorophore, that fluoresce only after special treatment have attracted the attention of researchers, as enzymes of high specificity are critical biomarkers in cancer, infectious diseases, inflammatory disorders, and so forth (Chyan and Raines, 2018; Marshall et al., 2020; Dai et al., 2021; Wu et al., 2022). One type of smart probes is optically silent until the peptides with quenchers are cleaved by a protease. Another type of smart probes, aggregation-induced emission (AIE) probes, display much stronger fluorescent emission with enhanced aggregation (Shi et al., 2012; Qian and Tang, 2017; Niu et al., 2020), after the enzyme processing. With other enhanced, targeted, or imaging strategies, the multifunction smart fluorescent probes have further improved sensitivity and selectivity for bioimaging systems.

This mini review intends to present new advances in multifunction enzyme-activated fluorescent probes. We describe the design of smart organic fluorophores and their imaging properties and discuss the existing challenges and future perspectives for the application of targeted organic fluorophores.

## 2 Multifunction Enzyme-Activated Fluorescent Probes

In the past decades, enzyme-activated fluorescent probes have been developed based on different enzymes specifically in tumor or other tissues and have established generalizable methods for imaging in cells and *in vivo* model systems (Huang et al., 2017). Although enzymes are used because of their binding properties and catalytic activity, there are still problems about selectivity, sensitivity, persistence, and stability, as well as the fluorescence intensity, diffusion, clearance, and limited emission wavelength of fluorescent probes. Efforts have been made to solve the problems by introducing multifunction to the fluorescent probes.

**FIGURE 1:**
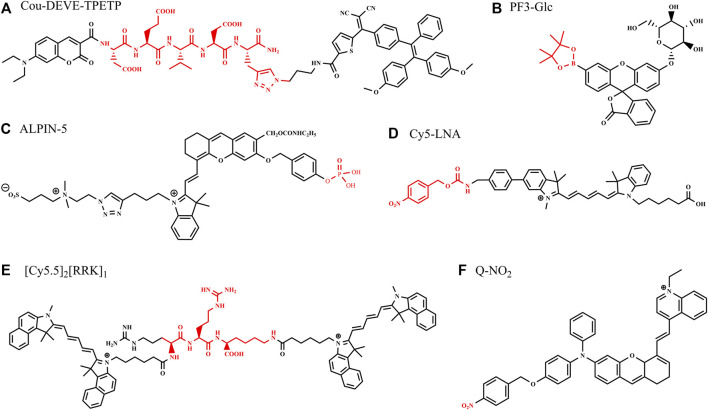
Structures of some bioimaging probes activated with single enzyme.

A novel two-signal turn-on fluorescent probe (Cou-DEVD-TPETP) was designed with the combination of fluorochrome (coumarin), Casp3 substrate (DEVD), and the quencher AIEgens (TPETP), as shown in Figure 1A. The probe itself is quenched as the energy transfer and dissipation of the acceptor energy through the free motion of AIEgens and is turned on by the processing of Casp3 with green coumarin–DEVD and the red TPETP residue. In this probe design, the energy quencher changes its role to a signal reporter upon enzyme activation, providing a second signal for imaging cell apoptosis (Yuan et al., 2016).

A dual enzyme-involved fluorescence-enhanced probe (PF3-Glc) was reported containing β-glucosidase (β-glc) and hydrogen peroxide (H_2_O_2_) trigger units (Odyniec et al., 2019), increasing fluorescence intensity by the unique cascade reaction *in situ*. β-glc catalyzes the hydrolysis of glycosidic bonds with the release of glucose and has been identified as a target critically involved with breast cancer growth and chemoresistance. PF3-Glc (Figure 1B) turned to be a slightly fluorescent mono-boronate fluorescein (PF3) after the presence of β-glc, as glucose released. Subsequently, the by-product H_2_O_2_ was formed from catalytic reactions with glucose oxidase (GOx), which resulted in classic H_2_O_2_-mediated boronate oxidation and the release of the highly emissive fluorophore, with an 80-fold increase in the fluorescence intensity at 510 nm.

**FIGURE 2:**
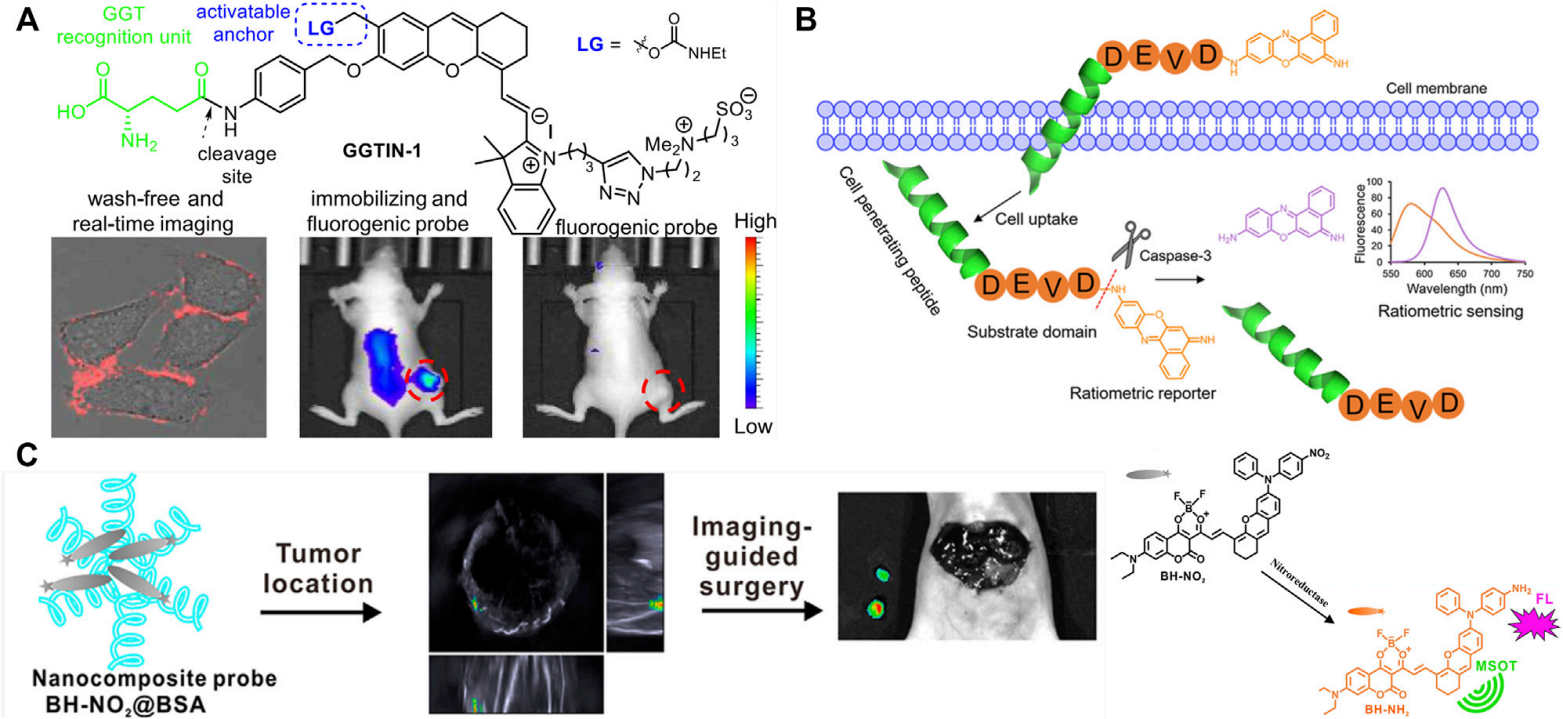
Design strategy and application of multifunction enzyme-activated probes with single enzyme. **(A)** Design of GGT-activated self-immobilizing fluorophore and its real-time imaging in mice [adapted with permission from Li et al. (2020b) Copyright © 2020, American Chemical Society]. **(B)** Design of Casp3-activatable cell-permeable fluorophore and its application on apoptosis imaging in HeLa cells [adapted with permission from Jin et al. (2021) Copyright © 2021, American Chemical Society]. **(C)** Design of dual-modal imaging probe and its application on imaging-guided surgery in mice [adapted with permission from Zeng et al. (2020) Copyright © 2020, American Chemical Society].

An activatable self-immobilizing fluorophore (GGTIN-1) was designed by merging quinone methide and a fluorogenic enzyme substrate (Figure 2A), activated by γ-glutamyl transpeptidase (GGT) for the *in vitro* and *in vivo* imaging (Li et al., 2020b). GGT is a type of cell membrane-bound enzyme, overexpressed specifically in cancer cells. This probe has three units, GGT recognition unit, cleavage site, and quinone methide (anchor) unit. GGTIN-1 is selectively activated by GGT, significantly increasing its fluorescence intensity at 714 nm. At the same time, activated fluorophores are covalently anchored at the site of interest (Figure 2A). More importantly, the use of this probe in U87MG tumor-bearing mice leads to much improved imaging sensitivity compared to regular fluorogenic probes because of this self-immobilizing ability. This group also developed an alkaline phosphatase (ALP)-activated imaging reagent (ALPIN-5, Figure 1C) covalently anchored at sites of activation with the fluorescence intensity at 710 nm, using NIR fluorophore itself as a quinone methide precursor (Li et al., 2020a)

Multifunction enzyme-activated and cell-permeable probes were reported to increase accuracy and penetrability of bioimaging (Ji et al., 2019; Jin et al., 2021; Zhu et al., 2022). Ac-Tat-DEVD-CV (Figure 2B) was designed by merging a cell-penetrating peptide and a fluorogenic enzyme substrate (Jin et al., 2021). The probe comprises a cell-penetrating peptide Tat, a caspase-3(Casp3) recognition sequence (Asp-Glu-Val-Asp, DEVD), and a fluorophore (cresyl violet, CV), measuring caspase-3 activity in living cells based on fluorescence change after caspase-3 activation. The Ac-Tat-DEVD-CV fluorescence emission wavelength changed from 582 to 628 nm as DEVD was cleaved by Casp3, and the ratiometric fluorescence signal (I_628_/I_582_) was used for monitoring Casp3 activity and apoptosis imaging in HeLa cells. By the introduction of lipophilic nitroaromatic moieties, fluorogenic probes (LNA-Cy5, Figure 1D) based on cyanine 5(Cy5) for identification of bacterial nitroreductase (NTR) have been reported to be cell-impermeable (Ji et al., 2019), where lipophilic nitroaromatic moieties also serve as caging groups decorating at the benzene unit or at the methine backbone of the Cy5 core. Catalytically reduced by NTR, the selected probe generates a rapid 10-fold fluorescence response at 657 nm and successfully images NTR in different live bacterial cells.

Some enzyme-activated probes are designed to offer multiple imaging modality to promote the application in disease monitoring, tumor imaging, intraoperative imaging, *in vitro* diagnostics, and point-of-care testing. Fluorophores are connected with nanomaterials or other contrast materials to realize multiple imaging modality with PET, CT, MRI, and PAI (Yan et al., 2019; Hu et al., 2021; Lei et al., 2022). For example, Gd-labeled nanoparticles coupled with Cy5 and cell-penetrating peptides were used for fluorescence and MR imaging (Olson et al., 2010). Based on the principle “light in and ultrasound out,” photoacoustic (PA) imaging can circumvent the main limitations of strong photon scattering and autofluorescence by tissues and resolve tissue chromophore distribution by identifying a sequence of photoacoustic images acquired at multiple illumination wavelengths, which is also called multispectral photoacoustic tomography. PA and fluorescence imaging can be constructed from the single fluorophore, offering more accurate self-verified information (Lavaud et al., 2020; Chen et al., 2021; Zhou et al., 2021). A set of peptide–dye probes is synthesized for PA and fluorescence imaging, and the imaging work demonstrates that the signal of the activated probe ([Cy5.5]_2_ [RRK]_1_, Figure 1E) was linearly correlated to the enzyme concentrate when imaged subcutaneously in mice (Moore et al., 2021). The peptide–dye conjugates were designed to undergo contact quenching via intramolecular dimerization, and the dyes are connected by peptide substrates. Employing trypsin as a model protease, proteolysis released single dye–peptide fragments, resulting in 330–4600-fold fluorescent enhancement and 5-fold PA enhancement with nanomolar sensitivity to trypsin.

Enzyme-activated AIE fluorescence imaging is an upcoming methodology to improve imaging accuracy. The endogenous enzymes, correlated with the severity of the diseases and the progression of the pathological conditions, are used for releasing probes from combined water-soluble state to hydrophobic AIEgens or from quenched state to fluorescent state, activating the probes and yielding a bright fluorescence. A series of research on enzyme-activated AIEgens for PA/fluorescence dual-modal imaging have been undertaken by the Wu group (Wu et al., 2019; Ouyang et al., 2020; Zeng et al., 2020; Huang et al., 2021). The NTR-activated probe BH-NO_2_@BSA is (Zeng et al., 2020) composed of the molecular probe (BH-NO_2_) and the carrier protein bovine serum (BSA). The BH-NO_2_ part has a diphenylxanthene group (electron donor) and a dioxaborininochromenone group (electron acceptor), and an aromatic nitro group on the donor side of strong electron-withdrawing capability is the fluorescence quencher, as well as the NTR-recognition moiety. With the carrier bovine serum affording water dispersibility and biocompatibility, BH-NO_2_@BSA could be readily taken up by the liver. In hepatic tumor cells, BH-NO_2_@BSA would be activated by the highly expressed NTR-reducing aromatic nitro group to diphenylamino group, endowing the activated probe with AIE feature. The activated probe (BH-NH_2_) exhibits an enhanced fluorescence at around 791 nm or 923 nm as well as a strong photoasorber for PA imaging. With dual-modal imaging NTR-activated probe, orthotopic liver tumors could preoperatively be precisely located by PA imaging, while tumor margins could accurately be defined by real-time intraoperative fluorescence imaging in the mice models (Figure 2C). Another NTR-activated probe Q-NO_2_ (Figure 1F) could detect and image sequential metastases from the orthotopic breast tumors to lymph nodes and then to the lung in two breast cancer mouse models, indicating the monitoring and tracking application on the cancer metastases and treatment efficacy during the chemotherapeutic course (Ouyang et al., 2020).

## 3 Multi-Enzyme-Activated Fluorophores

Enzyme activity can be used to process probes by cleaving the corresponding substrates in the fluorophores as well as to change their localization to be retained in cells within the particular microenvironment (Ofori et al., 2015). However, single enzyme could only provide limited selectivity between healthy and tumor tissues. Multi-enzyme activatable fluorophores strategies are proposed to comprehensively utilize the characteristics of different enzymes and improve the overall selectivity and sensitivity of bioimaging.

**FIGURE 3:**
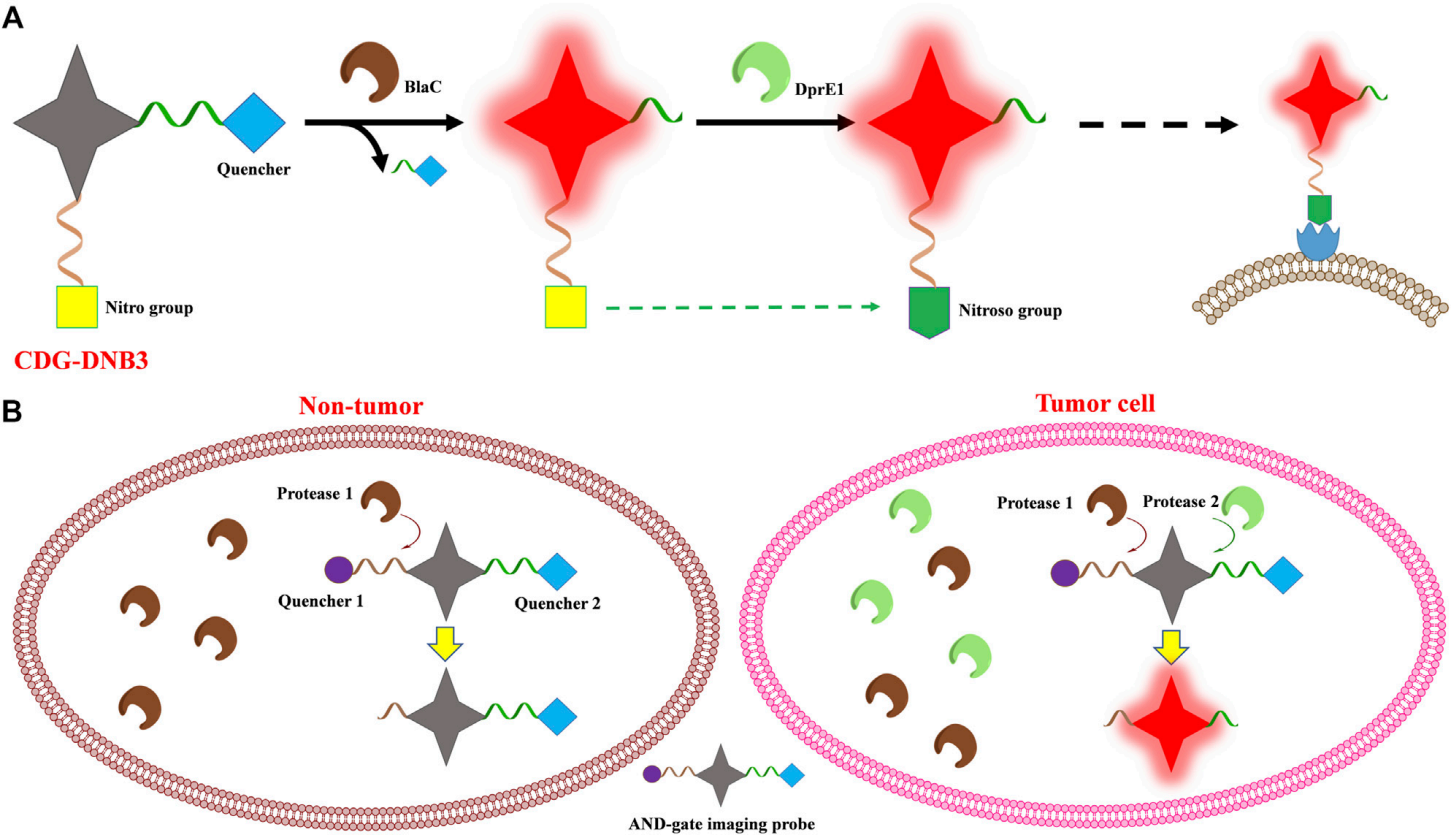
Scheme of the multi-enzyme-activated probe design strategy. **(A)** CDG-DNB3 and **(B)** AND-gate imaging probe.

A dual-targeting probe (CDG-DNB3) was developed for *Mycobacterium tuberculosis* (Mtb) imaging, targeting lactamase BlaC and decaprenylphosphoryl-β-d-ribose 2′-epimerase (DprE1), with the emission of 520 nm (Cheng et al., 2018). BlaC is a hydrolase naturally expressed in Mtb, and CDG-DNB3 combines a caged fluorescent reporter with the core structure of a β-lactam cephalosporin, which serves as a BlaC-sensing unit. CDG-DNB3 fluoresces as BlaC hydrolyzes the lactam ring to activate the fluorophore, resulting in high specificity for Mtb over other bacterial species. The Mtb essential enzyme DprE1 would reduce one nitro group to a nitroso derivative and covalently modify this nitroso to form a stable semi-mercaptal complex using the cysteine residue in the active site. Therefore, the DprE1-binding part of CDG-DNB3 would be covalently modified by DprE1, retaining the fluorophore in the cell to avoid signal diffusion. With the signal trapping by DprE1 and CDG-DNB3, one could discriminate live from dead Bacillus Calmette–Guérin (BCG) by fluorescence-activated flow cytometry analysis (Figure 3A). The dual-targeting probe also would provide the quantification function for rapid counting of Mtb within several seconds in combination using a self-driven microfluidic chip. Learning from the experience in the field of drug research, the dual-targeting probe approach realized the possibility to image Mtb at the single-cell level, through BlaC-mediated signal generation and DprE1-mediated signal retention, providing a new model for multi-targeted fluorophore design.

Widen et al. (2021) introduced multivariate “AND-gate” imaging probes with greatly enhanced selectivity and sensitivity, compared to single enzyme activated fluorophores. Multivariate AND-gate fluorophores combine substrates for multiple tumor-specific enzymes, requiring sequential processing by multi-enzymes to produce a fluorescent signal. A general AND-gate fluorophore contains a fluorescent reporter and multiple quenchers, with quenchers attached to the central fluorescent reporter through enzyme-sensitive linkages, and this probe would be fluorescence-activated only after all peptides have been cleaved by corresponding enzymes (Figure 3B). The glutamic acid (Glu) central linker is chosen to link the fluorochrome reporter and peptide substrates containing quenchers which are attached, respectively, to its free α-amine and carboxylic acids by diamino-alkyl linkers. Further to improve sensitivity and selectivity, the final fluorescent reporter will contain two free amine groups after peptide substrates are cleaved by corresponding enzymes, inducing lysosomal accumulation of the fluorophores. In this study, peptide substrates specifically processed by caspase 3 (Casp3) and the cysteine cathepsins (Cats) were used to compose DEATH-Cat probes to demonstrate the strategy. Cats, found in tumor-associated macrophages and normal tissues, have non-overlapping substrate specificities with Casp3, which is activated only in apoptotic cells. In this study, the activating enzymes Casp3 and Cats could exist in normal tissues, but they are only found together and activated in the tumor microenvironment, providing the high selectivity of the corresponding probes. Given the instability at the glutamine α-acid, (D)-Glu is used as the linker for stability of the AND-gate probes, and it is demonstrated in the cell imaging process. AND-gate probe DEATH-Cat-2 and its respective negative controls are evaluated in a 4T1 mouse model of breast cancer using the NIR imaging system, showing strong tumor accumulation. The brightest probe signal, always found at the intersection between macrophages and apoptotic cells with active Casp3, demonstrates that Casp3 and Cats are both active within tumor tissues.

To further certificate the AND-gate strategy, the homologous probe targeted other enzymes was designed. Similar to Casp3, FAPα is highly expressed in the tumor microenvironment, with higher proteolytic activity in a broad range of tumor types. New probe FAP-Cat targeted FAPα and Cats was synthesized with the corresponding substrates of FAPα and Cats according to the AND-gate strategy, resulting in a more concentrated signal at the outer margins of the tumor compared with previous results of DEATH-Cat-2 in a 4T1 mouse model of breast cancer. Meanwhile, the DEATH-Cat-2 probe produced a significantly brighter signal in a metastatic lung cancer model.

Robotic fluorescence-guided surgery with improved AND-gate probes DEATH-Cat-FNIR for a Vinci Surgical System was taken to resect of a primary subcutaneous mammary tumor and subsequently used probe fluorescence to assess the remaining subcutaneous tumor bed. Analysis of the H&E-stained tissues by a board-certified pathologist confirmed accurate detection of residual tumor cells by the AND-gate probes DEATH-Cat-FNIR after excision of a bulk tumor. After another robotic fluorescence-guided surgery to resect small metastatic cancer lesions in a metastatic lung cancer model, the resected lung tissues were imaged using the LiCor Pearl imaging system to confirm the probe selectivity of AND-gate probes DEATH-Cat-FNIR for metastatic lesions. The fluorescence signal of the resected lung section from the AND-gate probe also matched the location of tumor cells as determined by adjacent H&E-stained slides, indicating the ability of the AND-gate probe as a diagnostic indicator. Combining the selectivity of different enzymes, multivariate AND-gate imaging probes demonstrate a versatile platform for real-time intraoperative fluorescence imaging and tumor diagnosis.

## 4 Challenges and Outlook

Multifunction enzyme-activated fluorescent probes for bioimaging have been summarized in this mini review. Design strategies of multifunction fluorescence probes are sorted to single-enzyme-activated probes with more functions and multi-enzyme-activated probes listing the representative recent research work, involving fluorescence enhancement, enzyme immobilizing, activation reaction, multiple imaging modality, and multi-enzyme activation. The fluorescence imaging in cell or *in vivo* is also summarized to further evaluate the promising clinical applications.

The construction of enzyme-activated fluorescent probes based on the different strategies should be noted. Quenched fluorescence probes are activated by cleaving the quenching group (containing substrates) by the selected enzyme, and the remaining part of the fluorescence probes have the disadvantage of easy diffusion to other sites and excretion by the biological system. Therefore, the ability of *in situ* detection and long-term tracking of enzyme-activated fluorescent probes is influenced. Moreover, this type of fluorescent probes is prone to the aggregation fluorescence quenching (ACQ) phenomenon in high concentration in biological systems. However, it still has broad application scenarios of this strategy, as relatively simple in design and easy to implement. On the other hand, AIE fluorescent materials have very low fluorescence efficiency in dilute solvents and can achieve stable and highly efficient luminescence under aggregation or solid-state conditions, enabling *in situ* detection and long-term tracking of a particular target. Water-soluble AIE fluorescent probes avoid single-molecule diffusion and excretion during biological detection, providing an effective *in situ* detection.

Numerous examples of enzyme-activated fluorescence probes have been reported in the literature for selective visualization of specific markers in cells or tissues, but there is still room for improvement. A single-enzyme-activated probe may also show weak fluorescence in normal tissue, leading to instability, uncertainty, and diagnostic inaccuracy. There is a hard choice of the probe size because small molecule probes provide quick diffusion and deep penetration into living cells, while these probes and their enzyme-processed products suffer from easily diffusing ability across the cell membrane, leading to their limitation of real-time imaging and retention time. Multifunction enzyme-activated fluorescence probes provide possible solutions. Considering bioimaging requirement, there is a major trade-off between accessibility and wavelength. Longer wavelengths are better for tissue penetration and minimize tissue damage. Multimodality PA/fluorescence probes provide an additional solution to this problem. A series of overexpressed enzymes in tumor tissues have been utilized for enzyme-activated fluorescent probes. The successful design of a dual-enzyme strategy can significantly improve selectivity and accuracy to identify the location and size of specific tumor tissues, demonstrating fluorescence-guided efficient tumor resection, therapeutics, and even aiding the development of biomedicine.

Previous advance of enzyme-activated fluorescence bioimaging research lays the versatile and reliable foundation to broad applications in clinic-related fields. Enzyme-activated fluorescence probes are used to aid the diagnostic bioimaging, the fluorescence-guide surgery, and the evaluation of cancer treatment, as well as the research and development of anticancer and antibiotics drugs. It is hoped that this perspective will provide an insight into the enzymatic activated organic fluorescent probes for invasive, selective, and real-time bioimaging in cells or in model animals. There are a number of directions to explore in the future that could improve the performance of enzyme-activated fluorescent probes with more functions.
